# Hepatitis C-related knowledge and attitude among adults on probation in a large US city

**DOI:** 10.1186/s40352-024-00287-4

**Published:** 2024-07-11

**Authors:** Matthew S. Minturn, Kevin F. Kamis, David L. Wyles, Tracy Scott, Hermione Hurley, Scott J. Prendergast, Sarah E. Rowan

**Affiliations:** 1 Division of General Internal Medicine, Denver Health and Hospital Authority, 660 N. Bannock St. Ste. 7144, Denver, CO 80204 USA; 2 Public Health Institute at Denver Health, Denver Health and Hospital Authority, Denver, CO USA; 3 Division of Infectious Diseases, Denver Health Medical Center, University of Colorado School of Medicine, Denver, CO USA; 4 LGBTQ+ Health Services, Denver Health and Hospital Authority, Denver, CO USA; 5 Center for Addiction Medicine, Denver Health and Hospital Authority, Denver, CO USA; 6 Denver Adult Probation, Colorado Judicial Branch, Denver, CO USA

**Keywords:** Hepatitis C virus, HCV testing, HCV treatment, Adult probation

## Abstract

**Background:**

Hepatitis C virus (HCV) continues to cause significant morbidity and mortality within the US, and disproportionately impacts those involved with the criminal justice system. Despite this, knowledge and attitudes regarding HCV treatment among adults on probation have not been well studied. We conducted a cross-sectional survey of adults on probation accessing on-site HCV testing and linkage services at the adult probation department in Denver, Colorado. The survey assessed general knowledge of HCV and HCV treatment, as well as attitudes surrounding HCV treatment that might reflect medical mistrust. We used bivariate and multivariable logistic regression to identify factors associated with previous HCV testing, previous HCV treatment, and HCV antibody positivity at the time the survey was conducted.

**Results:**

A total of 402 participants completed all or a portion of the survey. 69% of the participants were cis-gender men; 29% were white, 27% were Black, and 30% were Hispanic/Latinx. Fewer than half of participants correctly identified that HCV infection is commonly asymptomatic (46%), that there is currently no vaccine that prevents HCV (19%), and that reinfection after treatment is possible (47%). Very few participants felt that side-effects (9%) or cost of treatment (10%) were barriers to care. Many participants believed that racial disparities exist in the treatment of HCV (59%). The belief that people who use substances are treated inequitably by health care providers was also commonly reported (35% of participants). Self-reported injection drug use and higher HCV-related knowledge were positively associated with previous testing for HCV. Higher HCV-related knowledge was positively associated with HCV antibody positivity at the time of survey completion, though the magnitude of the association was small.

**Conclusion:**

Interventions are needed to increase knowledge of HCV, to improve access to HCV testing and treatment, and to reduce bias associated with HCV and substance use within the probation population.

## Background

An estimated 2.4 million adults in the US were living with hepatitis C virus (HCV) infection in 2013–2016 (Hofmeister et al., 2019). The incidence of acute HCV infection (infection that is believed to have occurred within the previous 24 weeks) has been climbing since at least 2013 (Centers for Disease Control and Prevention, 2021) in association with the opioid epidemic and injection drug use in the US (Zibbell et al., 2018). Recent data from the CDC (2021) suggest that the prevalence of chronic HCV infection (infection that persists beyond 24 weeks from the time of infection) may be declining, likely due in part to the increasing availability of highly effective direct-acting antiviral medications for the treatment of HCV infection. Despite this, there remain significant barriers to treatment. One recent estimate of the HCV care continuum in the US suggests that only 52% of all those living with HCV were aware of their diagnosis and only 37% had received curative treatment (Chhatwal et al., 2019). Additionally, there remain significant disparities in treatment among subpopulations in the US. Among incarcerated people, for example, it was estimated that only 36% were aware of their diagnosis and only 4% had received curative treatment (Chhatwal et al., 2019).

Previous work has demonstrated that those who are uninsured or with public insurance, racial and ethnic minorities, and those who use drugs or alcohol are less likely to receive treatment for HCV (Nguyen et al., 2017; Sims et al., 2017; Wong et al., 2018; Zuckerman et al., 2018). Knowledge of HCV and attitudes surrounding HCV screening and treatment also may create barriers to receiving care. Surveys suggest that those with risk factors for HCV may not be screened because they do not believe they are at-risk, do not know where to receive screening, are worried about the cost of screening, or fear judgment from providers about behaviors that increase their risk (Barocas et al., 2014; Grannan, 2017; Jordan et al., 2013). Those with a known diagnosis of HCV may not receive treatment because they do not know that quick and effective treatment exists, do not recognize HCV as a serious health problem, do not know where or how to access treatment, are concerned about balancing competing priorities, or experience shame or guilt surrounding their diagnosis (Chen et al., 2013; Jessop et al., 2019; Jordan et al., 2013; Mittal et al., 2019; Nápoles et al., 2019; Skeer et al., 2018).

The prevalence of HCV infection is higher among those who are incarcerated, and particularly among incarcerated individuals with a history of intravenous drug use (Larney et al., 2013). However, those in prison or jail make up only 34% of the total US correctional population (Bureau of Justice Statistics, 2021). The largest subset of the US correctional population consists of those sentenced to probation (Bureau of Justice Statistics, 2021), where individuals are supervised in the community instead of being confined to prison or jail. Despite this, the epidemiology of HCV infection, the HCV care continuum, and knowledge and attitudes surrounding screening and treatment have not been well characterized in the adult probation population.

We partnered with the adult probation department in Denver, Colorado to better understand the landscape of HCV among those on probation. We conducted a retrospective study of a cohort of adult probation clients over a 25-month period to better characterize HCV prevalence and the HCV care continuum within this population (Kamis, Wyles, Minturn, Scott, McEwen, Hurley, Prendergast, Gunter & Rowan, 2022). We also trialed a voluntary HCV testing and care navigation program on-site at the probation department to determine if we could improve access to HCV testing and treatment among those on probation (Kamis, Wyles, Minturn, Scott, McEwen, Hurley, Prendergast & Rowan, 2022). Those who participated in the HCV testing and care navigation program were also asked to complete a survey to examine HCV-related knowledge and attitudes surrounding HCV testing and treatment. The results of this survey are described here.

## Methods

We offered on-site HCV testing and linkage-to-care services at the adult probation department in Denver, Colorado, U.S.A. Prior to testing, those interested in participating were asked to complete a short, self-administered survey that included demographic information, previous experience with HCV testing or treatment, and questions that assessed knowledge of and attitudes surrounding HCV testing and treatment.

### Setting

Denver Adult Probation provides supervision and related services to all adults sentenced to felony probation within the jurisdiction of the District Court of the State of Colorado’s 2nd Judicial District. They serve 4,500 to 5,000 clients in programs that have been designed to meet the client’s specific needs.

The Public Health Institute at Denver Health is a department within the Denver Health and Hospital Authority (henceforth referred to as Denver Health), an integrated public safety-net healthcare institution. The Denver Public Health Outreach Team performed study enrollment, survey collection, and HIV/HCV testing in a confidential area within the Denver Adult Probation building.

### Study population

All individuals aged 18 years or older engaged with one of the programs housed in the adult probation building were eligible to receive on-site HCV testing and care navigation services and were also eligible to complete the self-administered survey. The probation building housed pre-trial services and drug court in addition to the adult probation department, and individuals engaged with one of these programs were also eligible for inclusion. Those who reported at the time of enrollment that they were already engaged in HCV care were excluded.

All recruitment activities occurred on-site within the probation building. Flyer advertisements, word-of-mouth recruitment by probation staff, and verbal recruitment by testing staff were used to identify interested participants. All participants received a $25.00 pre-paid gift card in exchange for participation. Potential participants who declined to participate in the survey were still eligible for HCV testing and care navigation services free of charge.

The survey was administered on-site at the probation building prior to HCV testing. Participants completed the survey on tablets using a secure, HIPAA-compliant, web-based application called REDCap (Research Electronic Data Capture) (Harris et al., 2009). Research staff were present to assist with questions or any technical difficulties. All questions on the survey were optional.

### Measures

#### Participant demographics, previous HCV testing and treatment, and HCV risk factors

Participants were asked to report age, gender identity, sex assigned at birth, sexual orientation, race, ethnicity, highest level of education achieved, and type of health insurance. They also reported if they had ever been tested for HCV in the past and, if known, the results of their most recent previous HCV test. Those who had not been previously tested were asked to report why they had not been tested. Participants who reported a previous positive HCV test were asked to report any previous treatment, what type of treatment they received, and if treatment was completed. Those with a previous positive HCV test who did not receive treatment were asked to report why treatment was not started. Participants were asked to respond to one question about use of heroin, crack, cocaine, or methamphetamine within the past 12 months (including injection use, non-injection use, or both) and one question about condomless sexual intercourse within the past 12 months.

#### HCV-related knowledge and attitudes

Participants were asked to respond to five true/false questions and six Likert-scale items to assess HCV-related knowledge. They were asked to respond to five additional Likert-scale items to assess attitudes related to HCV testing/treatment and medical mistrust. The specific questions that participants were asked can be found in Table 1. These questions were developed and have been previously validated for assessing HCV knowledge and mistrust among those with HIV co-infection and for assessing mistrust of medical care in general (Allison et al., 2023; Bogart et al., 2011, 2021; LaVeist et al., 2000; Meyer et al., 2015). Response options for all true/false items were *true*, *false*, and *don’t know/not sure*. Responses to these items were converted to a numeric value of 1 for a correct response and 0 for an incorrect response. Responses of *don’t know/not sure* were considered incorrect. Response options for the Likert-scale items were *disagree strongly*, *disagree somewhat*, *don’t know/not sure*, *agree somewhat*, and *agree strongly*. Responses to these items were assigned a numeric value with a range of 1 through 5, with 1 representing *disagree strongly*, 3 representing *don’t know/not sure*, and 5 representing *agree strongly*.

**Table 1:** HCV Knowledge and attitudes among Survey participants

HCV Knowledge Items (True/False/Not Sure)	Correct Response		Total Responses		*n* (%) Correct		*n* (%) Incorrect ^c^
The majority of people who have hepatitis C do not have symptoms. ^a^	True		401		186 (46%)		68 (17%)
There are antiviral medications available to cure hepatitis C. ^a^	True		401		270 (67%)		27 (7%)
There is a vaccine to prevent hepatitis C. ^a^	False		401		76 (19%)		169 (42%)
People who are cured of hepatitis C, either naturally (by their own immune system) or with medical treatment can be infected with hepatitis C again. ^a^	True		400		187 (47%)		41 (10%)
Hepatitis C can cause liver damage. ^a^	True		399		295 (74%)		12 (3%)
**HCV Knowledge and Attitude Items (Likert Scale)**		**Total Responses**		***Mdn*** **(IQR) Score** ^**e**^		***n*** **(%) Agree or Strongly Agree**	
Hepatitis C treatment is too expensive and not available for most patients. ^a, d^		398		3 (2)		39 (9.8%)	
Even if a person who has hepatitis C doesn’t have symptoms, taking medication for it is a good idea. ^a^		401		3 (2)		173 (43.1%)	
Taking hepatitis C medications will keep people who are infected healthier longer. ^a^		401		4 (2)		221 (55.1%)	
Hepatitis C medications are too toxic or poisonous for most people. ^a, c^		399		3 (1)		34 (8.5%)	
The side effects of hepatitis C medications are too extreme for most people. ^a, d^		398		3 (0)		31 (7.8%)	
Hepatitis C medications have not been proven effective in curing hepatitis C. ^a, d^		401		3 (1)		38 (9.5%)	
People who take new medicines for hepatitis C are human guinea pigs for the government. ^b^		401		3 (2)		35 (8.7%)	
Rich patients receive better hepatitis C care than poor patients do. ^b^		400		3 (1)		79 (19.8%)	
People of color receive the same treatment for hepatitis C as White people. ^b, d^		399		3 (2)		164 (41.1%)	
People have been treated poorly or unfairly by doctors or healthcare workers because of their substance use. ^b^		399		3 (1)		140 (35.1%)	
Doctors and health care workers do not take seriously the medical complaints of people who drink alcohol or use drugs. ^b^		397		3 (2)		115 (29.0%)	

*Note. N* = 402. Survey participants had the option to skip any question; therefore, total number of responses varies among items.

^a^ Included in calculation of HCV knowledge score

^b^ Included in calculation of HCV medical mistrust score

^c^ Does not include responses of *don’t know/not sure*; however, such responses were considered incorrect when calculating the HCV knowledge score

^d^ Scale reversed for this question when computing knowledge or medical mistrust score

^e^ 1 = *disagree strongly*, 3 = *don’t know/not sure*, 5 = *agree strongly*

### Data protection

All data were password protected and stored on secure Denver Health servers accessible only to authorized members of the study team.

### Statistical analysis

Participant demographics were summarized using median and interquartile range or counts and percentages, as appropriate. Responses to true/false items were summarized by the number and percentage of correct responses. Responses to knowledge and attitude items assessed with a Likert scale were summarized using the median and interquartile range of the numeric scores for each item (1 = *disagree strongly*, 3 = *don’t know/not sure*, and 5 = *agree strongly*) as well as the number and percentage of those who reported agreement (*agree somewhat* or *agree strongly*) with the item.

HCV knowledge and medical mistrust scores were calculated from the true/false and Likert-scale responses for each participant. The numeric score for each Likert-scale item was divided by 5 to allow for more even weight of these responses alongside the true/false items. The 1 through 5 scale for some Likert items (those indicated in Table 1) was reversed so that higher scores consistently represented a greater degree of medical mistrust. The HCV knowledge score was calculated by summing the numeric values of responses to each true/false item and the knowledge-related Likert items. The medical mistrust score was calculated by summing the numeric values of each mistrust-related Likert item.

Bivariable and multivariable logistic regressions were separately performed for three outcomes: (1) self-report of previous HCV testing, (2) self-report of previously being offered treatment for HCV, and (3) positive HCV antibody test. The calculated HCV knowledge and medical mistrust scores were not included in the multivariable analysis because of significant interaction between these two predictors (Spearman’s ⍴ = -0.31) and between either knowledge or mistrust score and many other predictors in the model. All regressions were performed in R version 4.1.3 (R Foundation for Statistical Computing, Vienna, Austria). Prior to performing regression, incomplete responses were resolved by multiple imputation using the *MICE* package in R with 5 iterations as described by van Buuren and Groothuis-Oudshoorn (2011).

Previous studies have documented significant racial disparities in HCV testing or treatment and have suggested that medical mistrust contributes to this disparity (Bogart et al., 2021; Nguyen et al., 2017; Wong et al., 2018). Therefore, the interaction of HCV-related knowledge or medical mistrust on the relationship between race/ethnicity and self-report of previous testing in our study was further examined. Differences in knowledge and medical mistrust scores among white and non-white participants were assessed using Mann-Whitney tests. The roles of knowledge and mistrust were further explored with mediation analyses using the *mediation* package in R (Tingley et al., 2014) with nonparametric bootstrapping and 999 simulations.

### Ethics approval and consent to participate

This study was approved by the Colorado Multiple Institutional Review Board. Signed informed consent was obtained from all study enrollees. Consent emphasized that participation in the study would not affect participants’ legal proceedings and that information collected during this study would not be shared with anyone associated with their legal proceedings.

## Results

A total of 417 individuals enrolled in the study; 402 completed all or part of the survey. Each item on the survey was voluntary, therefore the number of responses to each survey item varies. Table 2 summarizes sociodemographic characteristics of survey participants. Most participants were male (69%) and had public insurance (86%). Just less than half of participants (44%) reported any type of drug use within the preceding 12 months, and 19% reported using injection drugs in this same period. Most participants were probation clients (87%); the remaining 13% of participants were participating in one of the other programs housed in the probation building (pre-trial services or drug court).

**Table 2:** Sociodemographic characteristics of Survey participants

Characteristic	Mdn (IQR) or *n* (%)
Age (*n* = 382)	38 (19)
Gender (*n* = 391)	
Cisgender male	269 (68.8%)
Cisgender female	118 (30.2%)
Other ^a^	4 (1.0%)
Race/Ethnicity (*n* = 391)	
White, non-Hispanic	115 (29.4%)
Black or African American, non-Hispanic	106 (27.1%)
Hispanic/Latinx, all races	119 (30.4%)
Multiracial, other	51 (13.0%)
Sexual orientation (*n* = 387)	
Heterosexual	329 (85.0%)
Gay or Lesbian	21 (5.4%)
Bisexual	35 (9.0%)
Other	2 (0.5%)
Health coverage (*n* = 390)	
Self-pay	19 (4.9%)
Public ^b^	335 (85.9%)
Private or employer-sponsored	16 (4.1%)
Other	20 (5.1%)
Attended college (*n* = 385)	178 (46.2%)
Self-reported drug use within the past 12 months (*n* = 384) ^c^	
None	217 (56.5%)
Injection	54 (14.1%)
Non-Injection	94 (24.5%)
Both	19 (4.9%)

*Note. N* = 402. Survey participants had the option to skip any question; therefore, total number of responses varies among items.

^a^ Includes transgender male/female, genderqueer, or “Other”.

^b^ Includes Medicaid, Medicare, CHAMPUS, Tricare, VA, or any other military-sponsored coverage.

^c^ Includes heroin, cocaine, or methamphetamine.

### HCV-related knowledge and medical mistrust

Table 1 summarizes responses to questions related to knowledge of HCV and medical mistrust. Most participants correctly identified that there are medications available to cure HCV (67%) and that HCV can cause liver damage (74%). Most participants also agreed that taking medication for HCV will keep people with HCV healthier longer (median score = 4; 25th percentile = 3; 75th percentile = 5). The greatest degree of medical mistrust seemed to be related to substance use, with participants tending to agree that people who use substances have been treated unfairly by healthcare providers because of their substance use (median score = 3; 25th percentile = 3; 75th percentile = 4).

### Odds of prior testing, prior treatment, and HCV antibody positivity

Three hundred ninety-two participants responded when asked if they had received previous HCV testing. Among those, 53 (14%) provided incomplete responses to other survey items that were resolved by multiple imputation prior to performing the bivariate and multivariable regressions. Repeating the regressions with removal of the incomplete records did not significantly alter the associations that were identified. One hundred seventy-eight (45% of those who responded) reported having received previous HCV testing. Table 3 summarizes factors associated with previous HCV testing.

**Table 3:** Odds of prior HCV testing among Survey participants by Sociodemographic Characteristic

Characteristic	Bivariate Regression			Multivariable Regression		
	*OR*	95% CI	*p*	*OR*	95% CI	*p*
Age	1.01	[1.00, 1.03]	0.11	1.01	[0.99, 1.03]	0.33
Gender						
Cisgender male						
Cisgender female	0.72	[0.47, 1.13]	0.15	0.62	[0.38, 1.01]	0.05
Other ^a^	3.31	[0.34, 32.5]	0.30	1.78	[0.14, 22.5]	0.65
Race/Ethnicity						
White, non-Hispanic						
Black or African American, non-Hispanic	0.76	[0.45, 1.29]	0.31	1.09	[0.60, 1.99]	0.78
Hispanic/Latinx, all races	0.58	[0.34, 0.97]	0.04	0.78	[0.43, 1.39]	0.39
Multiracial, other	0.42	[0.21, 0.84]	0.01	0.57	[0.27, 1.21]	0.14
Sexual Orientation						
Heterosexual						
Gay or Lesbian	0.73	[0.30, 1.82]	0.50	0.73	[0.28, 1.91]	0.52
Bisexual	0.95	[0.47, 1.92]	0.89	1.02	[0.46, 2.25]	0.96
*Health Coverage*						
Self-pay						
Public ^b^	1.91	[0.70, 5.15]	0.20	1.53	[0.52, 4.53]	0.44
Private, employer-sponsored	2.22	[0.56, 8.84]	0.26	1.84	[0.44, 7.72]	0.40
Other	1.91	[0.70, 5.15]	0.20	1.53	[0.52, 4.53]	0.44
Attended college	1.39	[0.92, 2.09]	0.11	1.41	[0.90, 2.19]	0.13
Self-reported drug use in the past 12 months ^c^						
None						
Injection	2.58	[1.39, 4.78]	< 0.01	2.65	[1.33, 5.29]	0.01
Non-injection	1.04	[0.64, 1.70]	0.87	1.01	[0.60, 1.70]	0.98
Both	1.88	[0.72, 4.88]	0.19	1.87	[0.69, 5.06]	0.22
HCV-related knowledge score ^d^	1.34	[1.19, 1.51]	< 0.01			
HCV-related medical mistrust score ^d^	0.90	[0.69, 1.17]	0.43			

*Note. N* = 392.

^a^ Includes transgender male/female, genderqueer, and “Other”

^b^ Includes Medicaid, Medicare, CHAMPUS, Tricare, VA, or any other military-sponsored coverage

^c^ Inclines heroin, cocaine, or methamphetamine

^d^ Excluded from multivariable analysis due to significant interaction with other predictors

Self-report of recent injection drug use was strongly and positively associated with prior HCV screening in both the bivariate (*OR* = 2.58, 95% CI [1.39, 4.78]) and multivariable (*OR* = 2.65, 95% CI [1.33, 5.29]) analyses. There was also a small positive association with higher HCV-related knowledge scores and prior HCV testing (bivariate *OR* = 1.34, 95% CI [1.19, 1.51]). There was no significant interaction between the HCV-related medical mistrust score and report of prior HCV testing.

Forty-two participants reported previous positive HCV testing and should have been eligible for treatment prior to enrollment. Among these, 10 (24%) reported having been previously offered HCV treatment, and only 3 of these reported having received at least some HCV treatment in the past. When logistic regressions were repeated with this subset of 42 participants using the same predictors listed in Table 3 for the outcome of having been offered previous HCV treatment, there were no relationships that met or approached statistical significance.

Four hundred one participants who completed the survey chose to undergo HCV testing at the time of survey completion. HCV antibody positivity following on-site testing was also examined as an outcome. Forty-nine (12%) of survey participants who underwent testing had a positive HCV antibody result. Relationships between sociodemographic characteristics and antibody positivity among all who received on-site testing are described separately (Kamis, Wyles, Minturn, Scott, McEwen, Hurley, Prendergast & Rowan, 2022). The analysis of survey responses described here identified a small positive association with higher HCV-related knowledge scores and antibody positivity (*OR* = 1.12, 95% CI [1.00, 1.25]). There was no significant association between HCV-related medical mistrust score and antibody positivity.

### Intersection of race and ethnicity with knowledge and mistrust

Further examination of the relationship between race and ethnicity and HCV-related knowledge and medical mistrust for the outcome of prior HCV testing revealed a small but statistically significant difference in HCV-related knowledge scores between white participants and those who identified with racial or ethnic minoritized groups (mean score 7.4 for white participants, 6.6 for those who identified with racial or ethnic minoritized groups, *p* < 0.01). Participants who identified with racial and ethnic minoritized groups also averaged slightly higher medical mistrust scores (3.0 vs. 2.8 for white participants), but not to an extent that reached statistical significance (*p* = 0.19). Mediation analysis revealed a very small but statistically significant mediating effect of HCV-related knowledge on the relationship between race and ethnicity and prior HCV testing (*OR* for average causal mediation effects = 0.96, 95% CI [0.92, 0.99]; *OR* for average direct effects = 0.93, 95% CI [0.83, 1.04]; *OR* for total effect = 0.90, 95% CI [0.79, 1.00]). Mediation analysis failed to reveal a significant mediating effect of medical mistrust on the relationship between race and ethnicity and previous testing.

## Discussion

Our study demonstrates that many of the same gaps in knowledge and trust related to HCV treatment present in other populations at risk for HCV are also present among adult probationers. Though there is some evidence of a shift toward greater knowledge and more positive attitudes toward HCV testing and treatment, continuing to look for ways to educate and engage adult probation clients in testing and treatment for HCV should be a priority for practitioners, public health professionals, and law enforcement professionals.

### HCV-related knowledge

Participants in our study demonstrated poor knowledge of some aspects of HCV infection and treatment. Many participants were not aware that there is currently not a vaccine to prevent HCV infection, that HCV infection is commonly asymptomatic, or that reinfection with HCV is possible after successful treatment. These knowledge gaps are similar to those identified in studies of populations with known HCV infection (Allison et al., 2023; Chen et al., 2013; Nápoles et al., 2019), those at higher risk for infection due to substance use disorders (Jessop et al., 2019; Jordan et al., 2013; Mittal et al., 2019; Skeer et al., 2018), those with HIV (Bogart et al., 2011), and those attending a Federally Qualified Health Center in a medically underserved neighborhood (Grannan, 2017). This suggests that gaps in HCV knowledge persist both within at-risk populations and among the general public. Educating probation officials about the importance of HCV testing and treatment, providing educational materials such as posters or pamphlets in probation facilities, and increasing availability of testing and care navigation services in probation facilities could improve knowledge and awareness of HCV among adult probation clients. Because probation clients live and work in the community, knowledge gained in the probation setting could quickly and easily spread to the community at-large.

It is encouraging that perceived side-effects and cost of treatment were not identified as being prominent barriers to treatment in our study. This is consistent with similar recent studies of those with HIV or HIV/HCV co-infection (Allison et al., 2023; Bogart et al., 2021); however, uncertainty among participants (responses of *don’t know* or *not sure*) in our study and the other studies was common. It is also possible that those with HIV (who are already taking daily medication that has been historically associated with negative side-effects and high cost) may be more comfortable with side-effects and cost of medication in general.

It is also encouraging that many participants in our study agreed that HCV treatment is warranted even if symptoms are not present, and that treatment of HCV will keep infected people healthier longer. Previous work has suggested that low perceived benefit of treatment and competing priorities (such as need for food or safe shelter) are significant barriers to seeking HCV testing or treatment (Balsom et al., 2023; Nápoles et al., 2019). However, those who completed the survey in our study had already indicated a desire for testing and care navigation services, so the opinions expressed in our survey may not represent the opinions of the larger probation population. There is compelling evidence to suggest that co-localization of HCV testing and treatment services with a syringe services program increases the likelihood of successful treatment (Eckhardt et al., 2022). Offering testing and care navigation services in the probation building, where participants were required to be for other purposes, may lessen the perceived burden of testing and treatment. On-site testing in the probation/parole setting has been described by others with encouraging uptake of testing; however, rates of off-site follow-up and treatment were low (Zaller et al., 2016). Rates of off-site follow-up and treatment after on-site testing in our own study were similarly low (Kamis, Wyles, Minturn, Scott, McEwen, Hurley, Prendergast & Rowan, 2022).

Increasing opportunities for testing and particularly for treatment in locations already frequented by those at-risk for HCV seems to be a promising approach to improve uptake of testing and treatment. This would likely require close partnership between probation departments and safety-net health systems to provide these services in non-traditional settings. Much of the counseling surrounding testing and treatment can be safely and effectively completed over the telephone or with secure videoconferencing. Providing time and space for probation clients to meet with a healthcare provider via a telehealth platform on-site at the probation department is one possibility that would allow co-location of testing and treatment services and could also be cost-effective and simple to implement. Further work is needed to determine the most effective implementation.

### Attitudes toward testing and treatment

We are encouraged by the positive association between prior HCV screening and history of injection drug use. A similar relationship has been described between higher HCV-related knowledge and having received treatment for a substance use disorder among adults with HIV and HCV co-infection (Allison et al., 2023). This suggests that efforts to target screening to those at increased risk for HCV acquisition due to drug use may be effective. Despite this, many participants in our study still agreed that people who use drugs are treated inequitably by the healthcare system or that their concerns are not taken seriously by healthcare providers. Healthcare providers and their institutions need to work harder to communicate in a more affirming way with people who use drugs and to reduce stigma associated with drug use and HCV infection.

Many participants in our study reported that race and ethnicity impact access to HCV treatment, consistent with findings reported in other, similar studies (for example, Bogart et al., 2021; Nguyen et al., 2017; Sims et al., 2017; Wong et al., 2018). We also identified a small but significant mediating effect of race and ethnicity on HCV-related knowledge as a predictor of previous HCV testing. This suggests that educational and outreach interventions targeting those who identify with racial or ethnic minoritized groups remains important. Interventions to encourage peer-peer support or education from peers may also be effective.

### Limitations

Our study has several limitations. Participants were recruited by convenience and there was no systematic sampling of the population of interest. Survey participation was limited to adult probation clients who agreed to HCV testing at a single site. For these reasons, the associations we identified may not be generalizable to the broader US adult probation population. Interactions likely exist between HCV-related knowledge, medical mistrust, and choosing to participate in HCV screening that could bias the associations we identified. Many participants had been previously tested for HCV and likely received some HCV education during past testing sessions that influenced their knowledge of or attitude surrounding HCV testing and treatment prior to participation in this study. Finally, this study was designed to be exploratory. Further work is needed to confirm the associations identified here or to establish causality within these associations.

## Conclusions

Individuals on court-ordered probation represent a unique group disproportionately burdened by HCV infection. Public health and law enforcement efforts are needed to develop interventions that provide HCV education and make accessing testing and treatment easier. Considering attitudes toward HCV and gaps in knowledge among those on probation may help inform these efforts. Culturally appropriate efforts that highlight the asymptomatic nature of many HCV infections, the lack of a vaccine against HCV infection, and risk of HCV reinfection after completing treatment could be particularly impactful among this population. Interventions that locate treatment services in areas frequented by those on probation may also be effective.

## Data Availability

The datasets generated and/or analyzed during the current study are not publicly available because they contain protected health information; deidentified datasets are available from the corresponding author on reasonable request.
